# Vitamin D3 as Potential Treatment Adjuncts for COVID-19

**DOI:** 10.3390/nu12113512

**Published:** 2020-11-14

**Authors:** Lucia Malaguarnera

**Affiliations:** Department of Biomedical and Biotechnological Sciences, University of Catania, 95124 Catania, Italy; lucmal@unict.it; Tel.: +39-095-4781272

**Keywords:** vitamin D, SARS-CoV2 immunopathology, immunomodulation, prevention

## Abstract

Severe acute respiratory syndrome coronavirus type (SARS-CoV2, also known as COVID-19), which is the latest pandemic infectious disease, constitutes a serious risk to human health. SARS-CoV2 infection causes immune activation and systemic hyperinflammation which can lead to respiratory distress syndrome (ARDS). ARDS victims are characterized by a significant increase in IL-6 and IL-1. Macrophage activation, associated with the “cytokine storm”, promotes the dysregulation of the innate immunity. So far, without vaccines or specific therapy, all efforts to design drugs or clinical trials are worthwhile. Vitamin D and its receptor vitamin D receptor (VDR) exert a critical role in infections due to their remarkable impact on both innate and adaptive immune responses and on the suppression of the inflammatory process. The protective properties of vitamin D supplementation have been supported by numerous observational studies and by meta-analysis of clinical trials for prevention of viral acute respiratory infection. In this review, we compare the mechanisms of the host immune response to SARS-CoV2 infection and the immunomodulatory actions that vitamin D exerts in order to consider the preventive effect of vitamin D supplementation on SARS-CoV2 viral infection.

## 1. Introduction

The new corona virus SARS-CoV2 has now become a major global concern. To date, 30 CoVs have been typified that can infect humans, mammals, and birds. The corona virus family has been categorized into four genera, the corona virus α, β, γ, and δ. Human CoV infections are generated by α- and βCoVs. The new SARS-CoV2 may have developed from the RNA recombination between existing corona viruses [1]. Human CoV infections mostly cause respiratory, gastrointestinal, hepatic, and central nervous system diseases. The symptomatology of SARS-CoV2 infection, including dry cough, fever, dyspnea, expectoration, fatigue, and myalgia resembles that of SARS-CoV and MERS-CoV [2]. SARS-CoV2 clinical manifestations vary considerably throughout the population. A large portion of subjects with SARS-CoV2 remain asymptomatic [3]. Several other patients have presented renal insufficiency, severe pneumonia, and bilateral ground-glass opacities on chest CT scans [4]. About 20–25% of SARS-CoV2 patients are affected by intestinal symptoms such as those observed in MERS-CoV or SARS-CoV [3]. In severe SARS-CoV2 infection subjects, the symptomatology may cause acute respiratory distress syndrome (ARDS), septic shock, multiple organ failure, metabolic acidosis, bleeding, and coagulation dysfunction, and even death [5]. The mortality rate is variable in the different populations and currently cannot be quantified. SARS-CoV2 spreads via droplets, close contact, aerosol, and even fecal-oral transmission can be a possible route of infection [6]. Asymptomatic subjects and patients in the incubation period can infect other persons, leading to exceptionally arduous procedures for detecting and isolating patients in order to contain epidemic spread [7]. On the basis of genome homology with SARS, an analysis of nucleic acid sequence within the spike protein receptor-binding domain (RBD) has revealed that SARSCoV2 used angiotensin converting enzyme (ACE)-2 and dipeptidyl peptidase (DPP)-4 as a cell receptor [8]. Similar to SARS-CoV and MERS-CoV, SARS-CoV2 infection leads to inflammation and immune activation and the release of proinflammatory cytokines, including interleukin 1 beta (IL-1β) and IL-6 [9]. Therefore, innate and adaptive immune responses exert an important role in protective or detrimental responses. In the last decades, many experimental evidences have indicated that vitamin D regulated both innate and adaptive immune responses through vitamin D receptor (VDR), which was expressed in immune cells, including naïve or activated CD4+ and CD8+ T cells, B cells, neutrophils, monocytes, macrophages, and dendritic cells [10]. It has been observed that patients with vitamin D deficiency (VDD) exhibited high levels of IL-6 and tumor necrosis alpha (TNF-α) and activated monocyte phenotypes [11]. Therefore, a successful immune response can be suitably regulated by the vitamin D endocrine system which balances inflammation versus anti-inflammation. In this review, we compare the mechanisms among the host immune response to the infection and the immunomodulatory actions of vitamin D in order to evaluate whether the causative agent SARS-CoV2 is an infection that takes advantage of vitamin D deficient patients, thereby affecting the course of the disease, and if vitamin D supplementation may be useful to prevent this disease.

## 2. Vitamin D3

**Figure 1:**
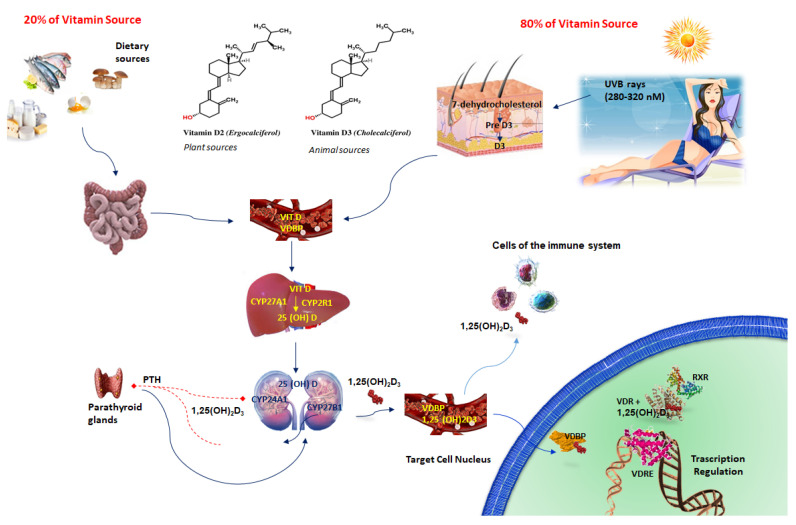
Vitamin D synthesis and mechanism of action. Vitamin D is biologically inactive and requires two enzymatic steps to become biologically active. Cutaneous 7-dihydrocholesterol is converted into preVitD_3_ after irradiation by ultraviolet light (UVB) from the sun. The first step occurs principally in the liver, where cholecalciferol is hydroxylated to 25-hydroxy-VitD (25(OH)D) or calcidiol, by the cytochrome P450 hydroxylase enzymes CYP27A1 and CYP2R1. Then, 25(OH)D is further converted, through a second hydroxylation in the kidneys by the mitochondrial cytochrome P450 enzyme (CYP27B1), into the active 1,25-dihydroxyvitamin D3 (1α,25(OH)_2_D_3_) or calcitriol. The activity of 1α,25(OH)_2_D_3_ is regulated by 24-hydroxylation (carried out by CYP24A1), which inactivates the hormone. Vitamin D activation takes place not only in the kidneys but also in other organs. Vitamin D is also activated locally by CYP27B1 in many cells including those of the immune system, where it influences a multitude of cellular functions. Active 1α,25(OH)_2_D_3_ binds the vitamin D receptor (VDR) and, afterwards, interacts with vitamin D response elements (VDREs) to modulate gene transcription. PTH, Parathyroid Hormone; RXR, Retinoid X Receptor; Vitamin D, VIT D; VDBP, Vitamin D Binding Protein

**Figure 2:**
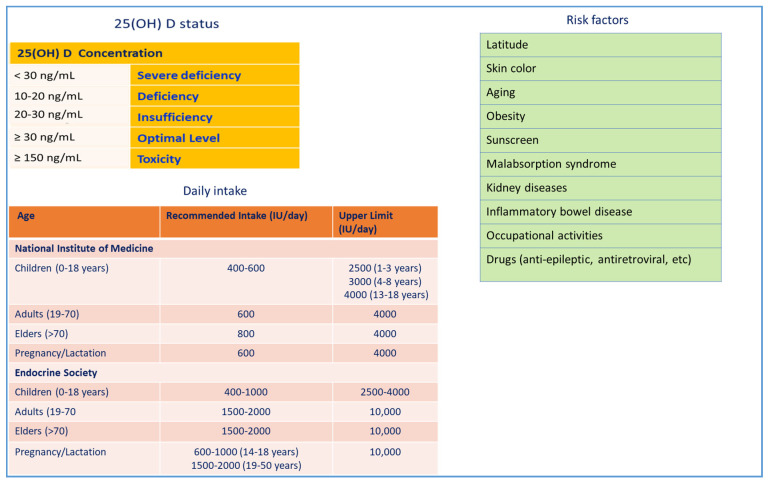
Vitamin D deficiency, daily intake, and risk factors. The international guidelines that focus on pleiotropic effects of vitamins recommend a target 25(OH)D concentration of 30 ng/mL. Vitamin D doses, with a range between 400-2000 IU/day, are recommended based on age, limited sunlight exposure, ethnicity, skin pigmentation, gastrointestinal absorption disorders, obesity, diabetes mellitus, liver and kidney disease, alcohol intake. Acute toxicity can occur using an excess dose of 10,000 IU/day of vitamin D, which results in serum 25(OH)D concentrations >150 ng/mL (>375 nmol/L). That level is more than the IOM-recommended UL of 4000 IU/day. Potential chronic toxicity would result from administering doses above 4000 IU/day for extended periods, i.e., for years, that cause serum 25(OH)D concentrations in the 50–150 ng/mL (125–375 nmol/L) range [13].

Vitamin D3 (1,25(OH)_2_D_3_) is a fat-soluble steroid with endocrine function. In addition to its conventional role in calcium homeostasis, it exerts other biological effects, such as regulation of cell proliferation, differentiation, and apoptosis, in numerous cell types and tissues. The two major forms of vitamin D, D2 (ergocalciferol) and D3 (cholecalciferol), are obtained in the skin when exposed to sunlight or, to a lesser extent, through diet and supplementation (Figure 1). VDD is a public health concern for all age groups around the world, both in industrialized countries where vitamin D is commonly integrated and in countries near the equator with remarkable UV radiation [12]. VDD may be due to limited sunlight exposure, skin pigmentation, black ethnicity, advanced age, low levels of dietary vitamin D intake, gastrointestinal malabsorption syndromes, liver and kidney diseases, obesity, diabetes mellitus, and alcohol ingestion [10]. Usually, vitamin D status is evaluated by serum 25-hydroxyvitamin D (25(OH)D) levels [13] (Figure 2).

## 3. Immunopathology of SARS-Cov-2

The initial site of SARS-Cov-2 infection is the lungs where the virus enters into the cells via the ACE2 receptor. However, it has been established that the SARS-Cov-2 infection triggered the innate and adaptive immune responses that caused a terrific inflammatory scenario resulting in tissue destruction and both local and systemic complications. Patients with severe SARS-Cov-2 showed significant reduced numbers of CD4+ T cells, CD8+ T cells, B cells, and natural killer (NK) cells [3,5] and a reduced number of monocytes, eosinophils, and basophils [14]. An increase in both neutrophil count and neutrophil-to-lymphocyte (NLR) ratio implies a further worsening of the disease and a poor clinical outcome [15]. In SARS-Cov-2 patients, the exhaustion markers, such as NKG2A, NK cells, and CD8+ T cells, are increased. In convalescent patients, CD4+ T cells, CD8+ T cells, B cells, and NK cells and the exhaustion markers get back to normal levels [16]. Patients with severe COVID-19 show higher serum levels of C-reactive protein, D-dimer and proinflammatory cytokines including IL-6, IL-1β, TNF-α, IL-2, IL-8, IL-17, G-CSF, GM-CSF, IP10, MCP1, and MIP1α (or CCL3) named as cytokine storm [3,5,14]. Furthermore, spleen atrophy and lymph node necrosis have been observed in deceased patients, revealing immune-mediated damage. Cytokine storm facilitates a severe pulmonary pathology, with massive infiltration of neutrophils and macrophages, resulting in a massive alveolar injury with the formation of hyaline membranes and a diffuse thickening of the alveolar wall [5]. In particular, overproduction of IL-6 is a valuable marker of poor outcome in SARS-CoV2 patients with ARDS. Therefore, cytokine storm by inducing a massive secretion of inflammatory mediators from immune cells, activating complement components, increasing oxidative stress, and reducing the expression of endothelial nitric oxide synthase, generates systemic inflammation and endotheliopathy, resulting in shock and tissue injury of the heart, liver, kidney, and multiple organ failure [17].

## 4. ACE2 Receptor and Host–Virus Interaction

**Figure 3:**
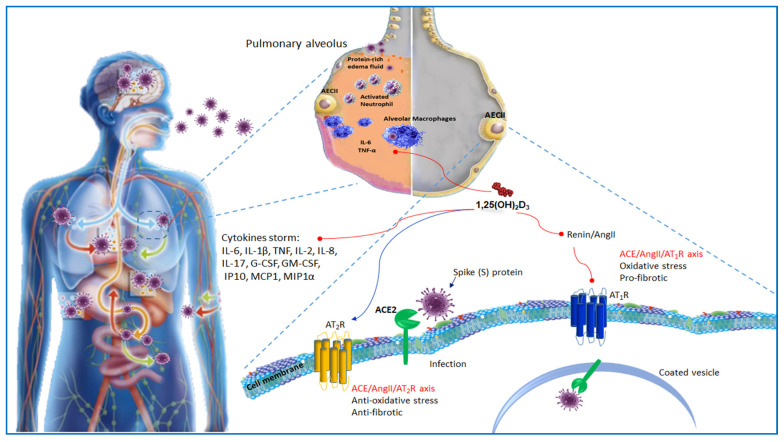
Host–SARS-CoV2 interaction and Vitamin D action. SARS-CoV2 interacts with the host ACE2 receptor using a spike protein S1 that facilitates the binding of the virion to the cell. Infection generates “cytokines storm”. ACE2-expressing alveolar epithelial type II cells (AECII) help corona viral replication. 1α,25(OH)_2_D_3_ induces ACE2/Ang-(1-7) axis and inhibits renin and the ACE/Ang II/AT_1_R cascade, and therefore prevents vasoconstriction, inflammation, cell proliferation, fibrosis, oxidative stress, and activates autophagy. ACE, Angiotensin Converting Enzyme; Ang II, Angiotensin II; AT_1_R, Angiotensin type 1 receptor; AT_2_R, Angiotensin type 2 receptor

The renin angiotensin system (RAS) is an endocrine system which plays physiological roles in electrolyte homeostasis, body fluid volume, and cardiovascular regulation in peripheral circulation. Renin is an enzyme produced in the kidneys. It acts on angiotensinogen (AGT), a liver precursor, to release an inactive decapeptide, angiotensin I (Ang I), which is later converted into angiotensin II by the angiotensin converting enzyme (ACE) present in the pulmonary capillaries. Genetic studies have identified the presence of homologous enzymes to ACE including enzyme 2 of ACE2. ACE2 catabolizes Ang II into Ang-(1-7), which by binding to mitochondrial assembly receptor (MasR) deactivates the deleterious actions of Ang II the effector peptide of RAS [18]. Consequently, to the binding to the angiotensin type 1 receptor (AT1R), Ang II enables vasoconstriction, inflammation, cell proliferation, fibrosis, increased renal sodium absorption, aldosterone, and arginine vasopressin (AVP) release. Moreover, Ang I and Ang II can bind angiotensin type 2 receptor (AT2). AT2, counteracting AT1 overstimulation, offsets the effects of the AT1 receptor inducing anti-inflammatory actions, vasodilation, cell apoptosis, and natriuresis [19]. SARS-CoV2 employs a spike protein S1 that facilitates the binding of the virion to the cell membrane cooperating with the host ACE2 receptor [20] (Figure 3). The ACE2 binding affinity of the SARS-CoV2 spike protein ectodomain has been demonstrated to be 10–20-fold higher than that of the SARS-CoV spike protein. This finding may explain the higher binding affinity of the SARS-CoV2 spike protein to the human receptor. The ACE2 receptor is expressed in numerous tissues including lungs, heart, luminal surface of intestinal epithelial cells, kidneys, and endothelium [20]. Hence, the clinical symptoms of respiratory injury, hepatic failure, acute kidney injury, or diarrhea are, at least in part, related to the widespread ACE2 expressing cells in these tissues. Furthermore, nCoV targeting ACE2-expressing cells affects various immune cells in various tissues, particularly, macrophages in lungs, liver, and stomach. Macrophages, enrolled by nCoV-targeted cells by means of the CD74/MIF interaction and additional signaling pathways during infection, may accomplish both defensive and destructive functions. Although ACE2 is modestly expressed in the lungs, it is the most susceptible target organ. About 83% of ACE2-expressing cells are alveolar epithelial type II cells (AECII). Therefore, these cells can act as a storage for viral irruption [21]. Gene ontology enrichment studies have proven that AECII expressing ACE2 showed an increment of multiple viral process-related genes, including regulatory genes for coronavirus processes, such as virus life cycle and viral assembly [22]. Therefore, AECII expressing ACE2 helps coronavirus replication. In addition, as receptors, the human coronaviruses exploit other peptidases including alanyl aminopeptidase (ANPEP), glutamyl aminopeptidase (ENPEP), and DPP4, which are the main genes correlated to ACE2 [23].

## 5. ACE2 Receptor and Vitamin D

ARDS, characterized by endothelial impairment and increased barrier permeability, is the most important cause of morbidity and mortality in patients with severe SARS-CoV-2. In a murine model of ARDS, ACE2 knockout mice developed a severe lung disease caused by increased vascular permeability and pulmonary edema as compared with wild mice [24]. Inhibition of the prorenin receptor decreased interstitial edema, hemorrhage, neutrophil number, and the level of non-proteolytically activated prorenin in the lung tissues of rats [25]. Therefore, since ACE2 can protect against acute lung injuries, its inhibition by SARS-CoV-2 could be closely linked with ARDS. Systemic infusion of Ang II promotes ALI [26]. Moreover, dysregulation of local and circulating RAS, together with increased expression of ACE/Ang II l and reduced levels of ACE2/Ang-(1-7), promoted ischemia-reperfusion-induced acute lung injury (ALI) in mice [27]. ACE2 overexpression reduced LPS-induced ARDS via the Ang-(1-7)/MasR pathway preventing extracellular signal-regulated kinase/nuclear factor kappa B (NF-κB) activation [28]. Several findings have indicated that 1α,25(OH)_2_D_3_ regulated RAS inhibiting renin biosynthesis [29]. A vitamin D-mediated pathway may avoid acute lung injury. It has been reported that 1α,25(OH)_2_D_3_ inhibited renin, ACE, and Ang II expression, and increased ACE2 levels in LPS-induced ALI. Hence, vitamin D reduced LPS-induced ALI by, at least partially, eliciting ACE2/Ang-(1-7) axis activity and inhibiting renin and the ACE/Ang II/AT_1_R cascade. VDR is significantly expressed in the lungs. It has been demonstrated that VDR inactivation resulted in deregulated stimulation of RAS. VDR and 1,25(OH)_2_D counteract fibrosis in lungs, liver, and kidneys [30,31,32] via the negative regulation of RAS and the inhibition of nuclear factor kappa B (NF-κB) and wnt/β-catenin [28,32]. In addition, VDR mediating this activity avoids sepsis-induced lung injury by hampering the angiopoietin-2-TEK receptor tyrosine kinase-myosin light-chain kinase pathway [33]. VDD is commonly seen in patients developing ARDS [34]. It is likely that VDD promotes lung fibrosis by activating RAS [35]. Consequently, RAS inducing fibrosis decreases lung function and compliance [36].

It has been observed that LPS-induced ALI in VDR-knockout mice was more severe, due to reduced occludin and zonula occludens-1 expression and the subsequent collapse of the alveolar epithelial tight junctions [37]. Vitamin D pretreatment decreases seawater aspiration-induced ALI by preventing inflammatory reactions, decreasing lung epithelial-endothelial barrier permeability, and modulating the RAS cascade. Paricalcitol, a vitamin D analog, protecting alveolar barrier function, alleviated LPS-induced ALI. These extraordinary effects occurred by inhibition of NF-κB and Ras, the homolog family member A/Rho kinase signaling pathways [38]. Inflammation may impair 1α-hydroxylase activity in the kidney, resulting in decreased production of 1α,25(OH)_2_D_3_ by inhibiting the PTH-stimulated conversion of 25(OH)D to 1α,25(OH)_2_D_3_ [39]. Interestingly, 1α-hydroxylase-deficient mice displayed enhanced activity of the intrarenal RAS that decreased following administering 1α,25(OH)_2_D_3_ [40]. In many disorders, the VDRA/VDR complex could mitigate RAS activity, reduce epithelial-to-mesenchymal transition, prevent apoptosis, decrease inflammation, trigger autophagy, control mitochondrial function, and induce immune tolerance through different signaling pathways. Experimental and clinical studies have indicated that pharmacological activation of VDR provided a tempting target for numerous inflammatory diseases [10].

## 6. The Host Immune Response COVID19 Infection

Through observing the remarkable similarities in genomic structure, in clinical and experimental data between the recent SARS-Cov-2 and previous SARS-COV and MERS-COV viruses, it is possible to hypothesize, step by step, how the host immune system acts with the SARS-Cov-2 virus. The innate immune system is a well-preserved defense apparatus essential for early recognition and restraining of pathogens and the consequent stimulation of the adaptive immune response. Once a coronavirus is inhaled, commonly, it binds to nonspecific receptors on the respiratory epithelium such as the adhesion molecule CEACAM1 [41]. It follows an endocytosis process which internalizes the virus and allows successive replication, transcription, and translation of new viruses that are, thus, released to infect new cells. The infected cells activate the innate immunity employing various pattern recognition receptors (PRRs), such as Toll-like receptors (TLRs) and RIG-I-like receptors (RLRs), and nucleotide-binding oligomerization domain (NOD)-like receptors (NLRs) that identify pathogen-associated molecular patterns (PAMPs). In spite of differences in each viral genome, replication schemes, and categories of PRRs activated, common signaling pathways are employed. 

## 7. Toll-Like Receptors

**Figure 4:**
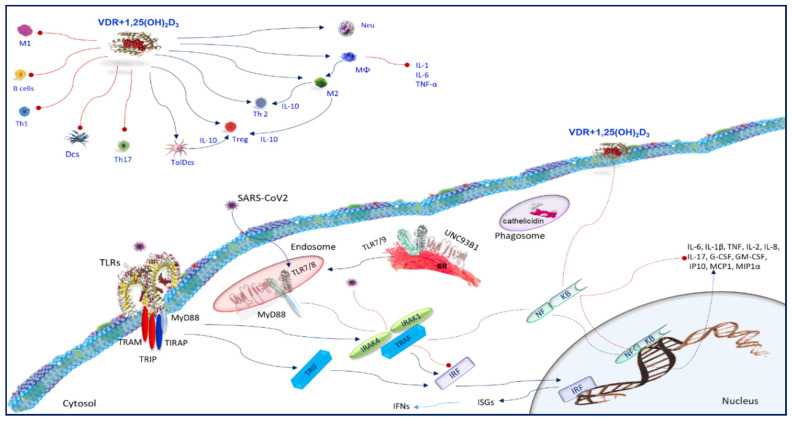
Host immune response COVID19 infection and Vitamin D function. Toll-like receptors (TLRs) are able to recognize viruses and carry them to the endosome. Different TLRs induce various biological responses via activation of MyD88, TIRAP, TRIP, and TRAM. MyD88 activates the nuclear factor kappa B (NF-κB) pathways to induce inflammatory cytokines production. Unc-93 homolog B1(UNC93B1) is essential for signaling of TLR3, TLR7, and TLR9. It interacts TLRs in the endoplasmic reticulum (ER) following viral infection. After a TLR is activated by the matching PAMP, MyD88 recruits IL-1 receptor-associated kinase (IRAK)-4, and then induces IRAK4 to activate other members of the IRAK, such as IRAK1 and IRAK2. Then, IRAK4 activates NF-κB and MAPKs downstream of MyD88. IRAKs interacts with TNF receptor-associated factor (TRAF) 6 and activates NF-kB. TRIF is an adapter protein of TLR3 and TLR4. TRIF-dependent pathways activate NF-kB and IFN receptors (IRFs). They induce the expression of various interferon-stimulated genes (ISGs), which reduce infection by their antiviral and immunomodulatory actions. SARS-CoV recognizes TLR7 and induces the proinflammatory cytokines such as TNF-α, IL-6, and IL-12. Coronaviruses inhibit type I IFN production and prevent the signaling downstream by inhibiting IRF3 nuclear translocation. 1α,25(OH)_2_D_3_ promotes TLR2 and TLR4. In response to TLR2/1 activation in human macrophages, 1α,25(OH)_2_D_3_ promotes innate immunity effectors and induces the synthesis of cathelicidin. MyD88, Myeloid differentiation primary response 88; TRAM, TRIF-related adaptor molecule; TIRAP, TIR domain-containing adapter protein; TRIP, TRAF-interacting protein.

Airway epithelial cells express TLRs and RLRs which perceive viruses. Ligands for TLRs and RLRs activate epithelial cells triggering a prompt immune response against viral attack [42]. In addition to the early infection of the epithelial cells, tissue-resident macrophages, intraepithelial dendritic cells (iDCs), which reside under the respiratory epithelium, internalize particles in the lumen of the airways by phagocytosis and macropinocytosis, thus, stimulating PRR and starting an immune response [43]. The TLRs belong to single-pass transmembrane receptors expressed in innate immune cells. They perceive efficiently a wide range of pathogen-derived molecules and influence host/pathogen interactions. The PAMPs detection by TLRs take place in cell membranes, endosomes, lysosomes, endocytolysosomes, and other sites of the cells [44]. TLRs identify proteins, lipoproteins, lipids, and nucleic acids of viral, bacterial, parasite, and fungal origins [44]. TLRs are either expressed on the cell surface (e.g., TLR2 and -4) or intracellularly (e.g., TLRs 3, 7, 8, and 9) mostly placed on the endoplasmic reticulum (ER) membrane [45] (Figure 4). TLRs recognize ssRNA (TLR7/8) or unmethylated CpG double-stranded DNA motifs (TLR9) of the viral genome, or the intermediary double-stranded RNA (TLR3) formed in the course of viral replication [46]. Furthermore, the TLR4 and TLR2 receptor complexes are able to recognize the viruses moving them to the endolysosome [47]. In particular, TLR7 which is one of PRRs recognizing ssRNA viruses through the identification of the viral RNA genome [48] is accomplished to detect SARS-CoV. TLR7 induces proinflammatory cytokines such as TNF-α, IL-6, and IL-12, recruits immune cells into the lungs, and promotes ARDS and lung fibrosis in the later stage [49]. Usually, coronaviruses affect the signaling downstream of IFNAR and inhibit type I IFN expression, which are linked with the disease severity [50]. Once type I IFN is induced, SARS-CoV prevents by ubiquitination and degradation RNA sensor adaptor molecules MAVS and TRAF3/6 inhibiting IRF3 nuclear translocation [51]. MERS-CoV also employs these procedures but also suppresses histone modification [51]. Hence, coronaviruses implement a mechanism to inhibit IFN signaling decreasing STAT1 phosphorylation [52]. The viral proteins modulating the host type I IFN response are structural proteins, such as M, N, or non-structural proteins such as ORF proteins, which are able to encode polyprotein replicases. It has been shown that papain-like protease (PLPro), encoded by ORF1, was involved in SARS-CoV replication exhibiting an antagonistic activity against type I IFN response. It involved IFN regulatory factor 3 (IRF-3) blocking IRF-3 phosphorylation and nuclear translocation [53]. Alternatively, PLPro impairs the stimulator of interferon gene (STING)-mediated signaling and as a result negatively regulates type I IFN induction [52]. PLPro interacting with the STING-TRAF3-TBK1 complex, reduces the ubiquitinated forms of STING, RIG-I, TRAF3, TBK1, and IRF-3. PLPro cooperates with these regulators of TLR signal pathways which characterizes the antagonistic mechanisms of TLR signal pathways (Figure 4). In SARS-Cov-2 infection, a similar scenario with a varying degree of immune interference may occur.

## 8. Vitamin D and Toll-Like Receptors

An important antiviral pathway in monocytes and macrophages takes into account the activation of TLRs [54] (Figure 4). Vitamin D metabolism in macrophages is associated with pathogen recognition [54]. In macrophages, TLRs induce upregulation of the VDR and CYP27B1 gene expression [55], initiating a signaling cascade essential to upregulate VDR and CYP27B1 which, in turn, induces the conversion of 25(OH)D to 1,25(OH)_2_D. This conversion could be a key phase to trigger the TLR pathway. It is well known that the binding of 1,25(OH)_2_D to the VDR enables expression of multitarget genes, which control the monocytes/macrophages function during infection. Macrophages internalize serum VDBP-bound 25(OH)_2_D_3_ from the extracellular fluid by endocytosis using 25(OH)_2_D_3_ as a substrate for upregulated CYP27B1. Since VDR is efficient in the presence of exogenous 1,25(OH)_2_D_3_, it can facilitate the ligation of the TLR2/1 heterodimer in macrophages and upregulated CYP27B1 [10,56]. Hence, extrarenal 1,25(OH)_2_D production is regulated both by TLR ligation and cytokine secretion, using a sophisticated crosstalk between different signaling pathways [56]. It is also possible that the expression of TLRs in various cell types, and the aptitude to react to a wide range of pathogens promotes additional TLRs or different PRRs to induce extrarenal expression of CYP27B1, and therefore locally synthesized 1,25(OH)_2_D can perform numerous actions in the immune response. The mechanism by which TLR ligation improves CYP27B1 expression involves NF-κB, JAK-STAT, and p38 MAPK pathways, and phosphorylation of the transcription factor C/EBPβ by p38 MAPK [57]. Cytokines, such as IL-1, IL-2, IL-4, IL-13, IFN-γ, and TNF-α, regulate CYP27B1 expression and vitamin D metabolism [56,57]. It is possible that the pathways involved in cytokine-regulated transcription of CYP27B1 are similar to the pathways employed by TLR ligation-induced regulation [58]. It has been demonstrated that the TLR8 ligands, CL097 and ssRNA40, induced a dose-dependent increase in CYP27B1 expression in macrophages [59,60]. In addition, 1α,25(OH)_2_D_3_ promotes TLR2 and TLR4, the receptor of interferon gamma (IFN-γ), or CD40 expression in monocytes [50], supporting a condition of hyporesponsiveness to PAMPs [10,61]. In human macrophages, following TLR2/1 stimulation, 1α,25(OH)_2_D_3_ activates innate immunity inducing antimicrobial peptides production [10]. Another interesting finding is that 1α,25(OH)_2_D_3_ regulates the TLR9-dependent production of IL-6 and increases NE/PAD4/COX-3/GAPDH, TLR7, and type I interferon (IFN) mRNA levels (Figure 4) [62]. It is possible that in individuals with adequate circulating levels of 25(OH)D, infection by SARS-CoV2 viruses following recognition by TLRs and cytokine production is able to intensify the levels of 1,25(OH)_2_D, which modulate the immune response to counteract the infections (Figure 4).

## 9. SARS-CoV2 and Immune Cells

Severe subjects with SARS-CoV2 show altered leukocytes count and NLR [14] The infiltrated immune cells in alveoli are mainly monocytes and macrophages [63]. Both helper T cells and suppressor T cells in patients with SARS-CoV2 are lower than the normal levels, whereas the amount of naïve helper T cells increases [14]. In addition, in patients with severe SARS-CoV2, the percentage of regulatory T cells (Tregs) is reduced [64]. Severe patients show increased levels of inflammatory cytokines and of infection-related biomarkers [64]. Similar to SARS-CoV or MERS-CoV infection, delayed type I IFN synthesis impairs the early viral control, generating an influx of hyperinflammatory neutrophils and monocytes/macrophages [65], which are the leading causes of lung dysfunction. The activation of these cells generates a worsening condition for the infected host that negatively impacts the outcome of the infection.

## 10. Vitamin D and Neutrophils

**Figure 5:**
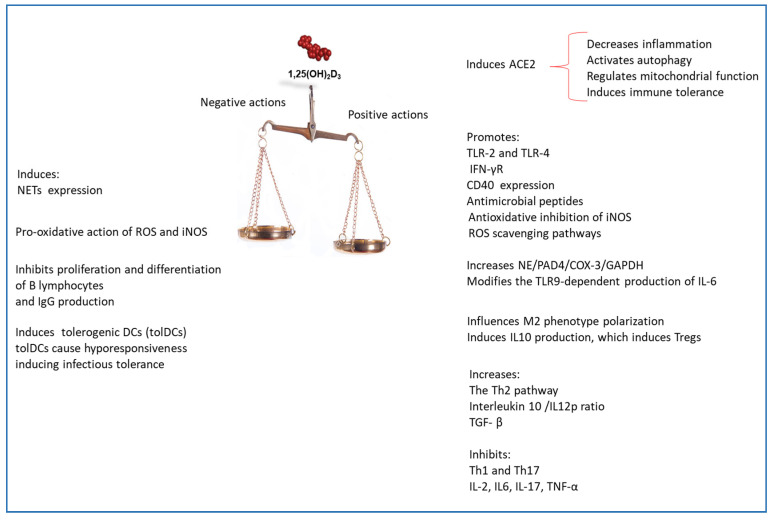
Positive and negative effects of vitamin D in immunomodulation against SARS-CoV2. DCs, Dendritic Cells; iNOS, inducible Nitric Oxide; NETs, neutrophil extracellular traps.

Often, SARS-CoV2 patients show an altered percentage of neutrophils [14]. Neutrophils are phagocytic cells that are specialized in early defense against invading pathogens [66]. They participate in innate immune responses restraining infectious diseases by means of microbicidal mechanisms such as phagocytosis, degranulation, and the delivery of neutrophil extracellular traps (NETs). NETs are extracellular strands of DNA in complex with histones and neutrophil granule proteins. They are released through the decondensation and spreading of chromatin and the adherence of various proteolytic enzymes, including neutrophil elastase (NE), myeloperoxidase (MPO), and peptidyl arginine deiminase 4 (PDA4). NETs have antibacterial activity, due mainly to the proteolytic enzymes that are able to destroy bacterial virulence factors. NETs are crucial for the host’s defenses and inflammation; they increase IL-1β and IFN-α production of activated neutrophils. In several SARS-CoV2 patients, high levels of NETs have been found [67]. 25(OH)_2_D_3_ plays an important role in modulating neutrophils activation and in preventing infections. It has been reported that 1,25(OH)_2_D_3_ was able to restrict the spread of pathogens, such as virus, by inducing NETs [68] and the expression of TLR7 and of IFN-α [62]. Nevertheless, excessive NET formation contributes to cytokine release and initiates a cascade of inflammatory reactions that facilitates micro-thrombosis generating a long-lasting organ damage of the pulmonary, cardiovascular, and renal systems [69]. Remarkably, these are three frequently affected organ systems in severe SARS-CoV2 [70]. These observations suggest that, in severe SARS-CoV2 stage, the administering of vitamin D3 could be ineffective or even deleterious (Figure 5).

## 11. Vitamin D and Monocytes/Macrophages

Macrophages are phenotypically and functionally heterogeneous cells. They exert a multitude of biological activities conditioned both by tissue microenvironment stimulation and cytokines signals [71]. This is because of the polarization of macrophages, which occurs as a result of stimulation with LPS and IFN-γ that generates classical activated macrophages (M1), or the generation of alternately activated macrophages (M2) as a result of stimulation with IL-4 and IL-13, or indirectly through the induction of Th2 cells, change their immune heritage [72]. M1 and M2 macrophages exert opposite activities, i.e., anti-inflammatory versus proinflammatory response, immunogenic versus tolerogenic activities, and tissue repair versus tissue destruction [72]. M1 macrophages are proinflammatory cells. They induce type 1/Th1/Th17 immune responses and synthesize a number of proinflammatory cytokines such as IL-1β, IL-6, IL-8, and TNF-α, and various cytotoxic molecules that induce the acquired immune response allowing the clearance of invading pathogens [73]. 1α,25(OH)_2_D_3_ affects macrophage polarization towards M2 phenotype [74] (Figure 4). M2 macrophages produce IL-10 which prevent the differentiation of M1 macrophages [75]. 1α,25(OH)_2_D_3_ modifies macrophage phenotype. In fact, reducing IFN-γ production and inducing IL10 synthesis prevents M1 macrophage differentiation [76]. M2 macrophages inhibit inflammation eliciting type 2/Th2 immune responses [77]. The airway occlusion, which occurs following SARS-CoV2 infection due to the massive occurrence of inflammatory cells, virus-infected cells, apoptotic cellular debris, and serum proteins, causes severe and potentially fatal ARDS [78]. In this context, the ability of M2 macrophages to phagocytose infected cells and apoptotic cellular debris, improved by the action of 1α,25(OH)_2_D_3_, could help to achieve the clearance of infection and the resolution of inflammation

## 12. Vitamin D and AMPs Induction

The 1,25/VDR/RXR complex enhances, in the macrophages, chemotactic and phagocytic activity and simultaneously triggers the transcription of antimicrobial peptides (AMPs) in several cell types (Figure 3). In macrophages, TLRs activation stimulates cathelicidin antimicrobial peptide (CAMP) expression through a vitamin D-dependent pathway [79]. The 1,25(OH)_2_D-VDR complex binds VDRE in the promoter of the cathelicidin gene enhancing hCAP-18 production [80]. The production of CAMP mediated by vitamin D increases antimicrobial activity against pathogens increasing phagosome formation and by direct action of cathelicidin leucin-leucin 37 (LL-37) [79]. This induction can be one of the mechanisms by which vitamin D improves innate immunity toward respiratory infections. LL-37 stands in epithelial cells, neutrophils, monocytes/macrophages, NK cells, B cells, and γδ T cells [81], and can be produced by respiratory epithelial cells of the airway surface. It represents the first line of defense against pathogens [82]. LL-37 modifies cytokine production enhances TLR3 signaling. Moreover, repressing directly viral particles directly, reduces the spread of infection and of infected epithelial cells and exerts an antiviral activity against both enveloped and non-enveloped viruses [83,84,85]. In monocytes, TLR activation induces defensin beta 4 gene (DEFB4) expression via the synergistic action of IL-1β and VDR pathways [86]. Interestingly, a VDRE is located in the proximal promoter region of DEFB4 gene, certainly, it allows the upregulation of β-defensin 2 expression under 1,25(OH)_2_D stimulation [80]. This is an additional antimicrobial peptide, which, as well as LL-37, promotes chemotaxis of immune cells and prevents viral infection [87].

## 13. Vitamin D and ROS and iNOS Generation

The oxidative burst has beneficial antiviral effects [88]. Nevertheless, aberrant induction of the oxidative burst is associated with pathophysiology and tissue damage [89]. In monocytes and macrophages, the generation of inducible nitric oxide synthase (ROS) and inducible nitric oxide synthase (iNOS) is mediated by PI3K signaling pathway. Interestingly, an important signaling pathway regulated by 1α,25(OH)_2_D_3_ is the class III phosphatidylinositol 3-kinase complex (PI3KC3) [90]. Therefore, in this way, 1α,25(OH)_2_D_3_ controls redox homeostasis in both pro-oxidative ROS and iNOS inductions to boost the antiviral response [79], and antioxidative inhibition of iNOS and induction of ROS scavenging pathways to prevent immunopathology [79]. 

## 14. Vitamin D and Autophagy 

Autophagy is an important process in the immune system, being a host defense tool against viral infections [91]. It is necessary to remove damaged proteins and organelles. In human monocytes, 1α,25(OH)_2_D_3_ induces the maturation of autophagosomes and autophagolysosomes using a hCAP18/LL-37-mediated pathway [92]. 1α,25(OH)_2_D_3_ induces autophagy by regulating multiple associated pathways, such as Bcl-2, mammalian target of rapamycin (mTOR), class III phosphatidylinositol 3-kinase complex, and cathelicidin production. Consequently, it enhances clearance of viruses and viral constituents [93]. Vitamin D triggering autophagy in macrophages could inhibit replication of SARS-CoV2. VDR and adequate levels of vitamin D could be associated with a natural resistance to SARS-CoV2 [94]. This may result from the upregulation of anti-inflammatory IL-10 and induction of defensin in mucosa of SARS-CoV2-exposed individuals 

## 15. Adaptive Immune Response

The humoral immune response by producing neutralizing antibodies protects the host by limiting infections in the later phase and preventing reinfections. In SARS-CoV, both T and B cell epitopes have been mapped for the structural proteins, S, N, M, and E protein [95]. Th1 type immune response plays a leading role in the adaptive immunity to viral infections. Cytokines, produced by antigen-presenting cells (APCs), directs T cell responses. T helper cells coordinate the global adaptive response, whereas cytotoxic T cells are indispensable in removing viral infected cells. In MERS-CoV infection, an early increase in CD8+ T cells is associated with disease severity. It has been observed that, in convalescent patients, Th1 cells were prevalent [96]. Th1 type response is crucial for an efficient control of SARS-CoV and MERS-CoV. In a murine model, airway memory CD4^+^ T cells specific for conserved epitope protect against fatal complications and cross-react with SARS-CoV and MERS-CoV [97]. A similar immune response may also be possible for SARS-CoV-2. In these viral infections, CD8^+^ T cell responses is more pronounced than CD4^+^ T cell responses. Therefore, CD8^+^ T cell reaction should be carefully monitored to prevent the occurrence of lung pathology. 

## 16. Vitamin D and the Adaptive Immune Response to Respiratory Pathogens

Vitamin D modulates the adaptive immune response, and since it influences the antigen presentation, it acts as a bridge between innate and adaptive immunity. 1α,25(OH)_2_D_3_ inhibits proliferation and differentiation of B lymphocytes and IgG production, as well as proliferation of Th1 cells and their cytokines production, whereas it increases the Th2 pathway [10]. Low 25(OH)D levels change the normal Th1 and Th2 cytokine balance, causing an increase in Th1 cytokine expression. Indirectly, 1α,25(OH)_2_D_3_ affects T cell responses influencing the dendritic cells (DCs) phenotype [98]. DCs are effective APCs affecting lymphocyte activation and inducing the adaptive immune response. DCs express VDR, CYP27A1, and CYP27B1, therefore, they produce 1α,25(OH)_2_D_3_ [99]. Nonetheless, human monocyte-derived DCs convert 25(OH)D to 1α,25(OH)_2_D_3_ in a reduced quantity as compared with macrophages, probably because DCs express a truncated CYP27B1 transcript that can result in a deficiency in vitamin D activation [100]. The addition of 1α,25(OH)_2_D_3_ prevents DC differentiation, maturation, and antigen presentation; decreases the co-stimulatory molecules CD40, CD80, and CD86 [101]; reduces CD1a, MHC class II, and abolishes the chemotactic response to CCL4 and CCL19 [102]. The primary function of DCs is to trigger T cell response and, as a result, the effect of 1α,25(OH)_2_D_3_ on DCs has a strong impact on T cells. In vitro, 1α,25(OH)_2_D_3_ induces a stable maturation-resistant tolerogenic phenotype and increases interleukin 10 (IL-10)/IL-12p70 ratios. Administering of an antigen along with 1,α25(OH)_2_D_3_ can induce antigen-specific tolerogenic DCs (tolDCs) [103]. They become able to induce infectious tolerance, changing the activities of other proinflammatory mature DCs, inducing antigen-specific regulatory T cells (Tregs), and causing the maintenance of the tolerogenic response [104]. Even if cultured with committed T cells, tolDCs cause hyporesponsiveness, inhibit T cell proliferation, and decrease IFN-γ production [105]. The increased IL-10 production induces Tregs that, in turn, secrete more IL-10, TGF-β, and CCL22 [61]. In addition, 1α,25(OH)2D3 is directly capable of modifying T cell differentiation and cytokines produced. Production of IL-2, IL-17 IL-21, IFN-γ, and TNF-α, are all inhibited [106] and IFN-γ prevents macrophage activation, consequently, decreasing antigen presentation and the recruitment of other T cells [107]. The direct decline of Th1-priming cytokines further directs T cell differentiation towards a Th2 phenotype. In addition, 1α,25(OH)_2_D_3_ upregulates the Th2-specific transcription factors GATA-3 and c-maf, enhancing IL-4, IL-5, and IL-10 synthesis [108], reducing Th17 cells and IL-17 production. Th17 cells are a CD4^+^ T subset, and by synthesizing IL-17, they trigger an inflammatory response dominated by neutrophils. Th17 cells proliferation is induced by signals mediated by IL-6 IL-21, IL-23, TGF-β, and by retinoic acid-related orphan nuclear receptor (RORγT), which is a lineage-specifying transcription factor. However, on the basis of the cellular studies on the effects of vitamin D in Th2 cells [109] concerning both and increase [108] and reduction in IL-4 synthesis, the mechanisms behind any potential beneficial role of vitamin D are yet unclear and conflicting [110]. In addition, the finding shows that 1α,25(OH)_2_D_3_ downregulates DC-derived Ox40L, which is necessary for Th2 priming, thus, resulting in a reduced Th2 cytokine response in CD4+ T cells [111], and contradicts the notion that vitamin D induces the phenotype of T cells towards a Th2 one. The decline of Th1 immunity, which has been previously described [10], could suggest a lower immune response to pathogens, incompatible with the evidence showing an improved response to respiratory tract infections after vitamin D supplementation. Finally, studies have shown that, in lymphocytes, the administering of vitamin D exerts direct effects, whereas, in DCs, to exercise its immunomodulatory effects, the conversion of the inactive metabolite 25(OH)D in the active 1α,25(OH)_2_D_3_ is necessary [112]. This suggests that cellular stimulation with different vitamin D metabolites may elicit different responses. Therefore, while vitamin D is an immunomodulatory molecule with a wide range of effects, the specific mechanisms are, at this time, vague. In addition, the conflict among results reported adds uncertainties to its actions. Additionally, vitamin D modulating T cell responses is able to exert direct effects on B cells [113]. Human B cells also express VDR and CYP27B1, which are upregulated upon activation. Therefore, B cells are susceptible to 1α,25(OH)_2_D_3_ stimulation. It is able to inhibit proliferation, plasma-cell differentiation, and immunoglobulin secretion, and by inducing B cell apoptosis it is able to prevent memory B cell generation [114]. 

## 17. Conclusions

There is emerging evidence revealing the promising role of vitamin D in preventing cytokine storm and, consequently, determining outcomes of SARS-Cov2 [115,116,117,118,119,120,121,122,123,124,125,126,127,128]. Vitamin D insufficiency has been shown to be related to latitude, obesity, diabetes, hypertension, ethnicity, and sex, and it is a condition associated with the increased susceptibility for SARS-Cov2 infection and mortality. It has also been suggested that the different gender-related susceptibilities involve testosterone levels associated with vitamin deficiency in men [118]. In this review, we analyzed the immune response of the host to an SARS-Cov2 infection, also referring to the knowledge of the immune response against MERS-Cov and SARS-Cov2 given the high homology that these viruses show. In parallel, we evaluated in which repertoire of effector cells and molecules of the immune response vitamin D could intervene to counteract against SARS-COV2 infection. This examination highlighted the extreme complexity of mechanisms used by vitamin D for modulating the immune response following a viral invasion (Figure 5). Some clinical trials revealed that vitamin D supplementation was effective to prevent infection both in the early and in the hyperinflammatory stage of the disease, since it modulated efficaciously the immune response against SARS-COV2 [125,126,127]. However, following an in-depth analysis of the mechanisms involved in the immune response conducted in this review, it can be noted that vitamin D, in severe SARS-CoV2 stage, should be administered with caution because it could stimulate or inhibit some cellular functions that could induce infectious tolerance (Figure 5). The proposed guidelines for the treatment of COVD19 recommended the use of glucocorticoids. Therefore, the suggested treatment with glucocorticoids in combination with vitamin D supplementation [127] could be an interesting perspective to be taken into account. Since vitamin D has multiple cellular and intracellular targets, additional studies are needed to determine the consequences of the interaction of vitamin D in the immune-response against SARS-CoV2 in order to achieve a significant vision into prophylactic and therapeutic strategy for the prevention of this viral infection.
